# Anaemia management with subcutaneous epoetin delta in patients with chronic kidney disease (predialysis, haemodialysis, peritoneal dialysis): results of an open-label, 1-year study

**DOI:** 10.1186/1471-2369-10-5

**Published:** 2009-02-25

**Authors:** Ulrich Frei, Jonathan TC Kwan, Bruce S Spinowitz, The Epoetin Delta 3002 Study Group

**Affiliations:** 1 Department of Nephrology and Medical Intensive Care, Campus Virchow-Klinikum, Charité-Universitätsmedizin Berlin, Berlin, Germany; 2 Medical Lead for Renal Services, SW Thames Renal & Transplantation Unit, Epsom & St Helier University Hospitals NHS Trust, St Helier Hospital, Carshalton, Surrey, UK; 3 Weill Cornell Medical College, New York, NY, USA

## Abstract

**Background:**

Anaemia is common in patients with chronic kidney disease (CKD) and can be managed by therapy with erythropoiesis-stimulating agents (ESAs). Epoetin delta (DYNEPO^®^, Shire plc) is the only epoetin produced in a human cell line. The aim of this study was to demonstrate the safety and efficacy of subcutaneously administered epoetin delta for the management of anaemia in CKD patients (predialysis, peritoneal dialysis or haemodialysis)

**Methods:**

This was a 1-year, multicentre, open-label study. Patients had previously received epoetin subcutaneously and were switched to epoetin delta at an identical dose to their previous therapy. Dose was titrated to maintain haemoglobin at 10.0–12.0 g/dL. The primary endpoint was mean haemoglobin over Weeks 12–24. Secondary analyses included long-term haemoglobin, haematocrit and dosing levels. Safety was assessed by monitoring adverse events, laboratory parameters and physical examinations.

**Results:**

In total 478 patients received epoetin delta, forming the safety-evaluable population. Efficacy analyses were performed on data from 411 of these patients. Mean ± SD haemoglobin over Weeks 12–24 was 11.3 ± 1.1 g/dL. Mean ± SD weekly dose over Weeks 12–24 was 84.4 ± 72.7 IU/kg. Haemoglobin levels were maintained for the duration of the study. Epoetin delta was well tolerated, with adverse events occurring at rates expected for a CKD patient population; no patient developed anti-erythropoietin antibodies.

**Conclusion:**

Subcutaneously administered epoetin delta is an effective and well-tolerated agent for the management of anaemia in CKD patients, irrespective of dialysis status.

**Trial registration:**

http://www.controlled-trials.com ISRCTN68321818

## Background

Chronic kidney disease (CKD) is a prevalent and increasing health concern worldwide, and anaemia is a frequent complication of CKD [1,2]. The main cause of anaemia in patients with CKD is insufficient synthesis of erythropoietin by the damaged kidney. Since the 1980s, erythropoiesis-stimulating agents (ESAs) have been used to treat CKD-associated anaemia. Anaemia management with ESAs is a successful and well-tolerated therapy that has been demonstrated to improve quality of life and positively influence outcomes [3-6].

Until now ESAs have all been recombinant erythropoietins produced in Chinese hamster ovary (CHO) cell lines, however a new epoetin, epoetin delta (DYNEPO^®^, Shire plc), is the only one produced in a human cell line [7,8]. Epoetin delta has an identical amino acid structure to endogenous human erythropoietin, as is also the case for epoetin alfa and epoetin beta [7]. However differences in glycosylation profile between epoetin delta and the CHO-cell-derived epoetins have been demonstrated, such as lower levels of certain non-human carbohydrate residues (N-glycolylneuraminic acid) in epoetin delta [8].

Phase II clinical trials with epoetin delta demonstrated that it is effective in increasing haemoglobin levels when administered intravenously (three times per week) to haemodialysis patients [9] and subcutaneously (twice per week) to CKD patients not on dialysis [10]. A 24-week, phase III trial in haemodialysis patients comparing epoetin delta and epoetin alfa, administered intravenously three times per week, demonstrated equivalent efficacy between the two agents [11]. An open-label extension to the study showed continued efficacy of epoetin delta in this haemodialysis population throughout the 52 weeks of study [12]. Efficacy of epoetin delta in a subgroup of predialysis patients has also been previously described [13]. Epoetin delta was well tolerated in all of these phase II and phase III studies. Additionally, phase I and II studies demonstrated that the half-life of subcutaneously administered epoetin delta is similar to the half-life of epoetin beta [14], an agent that can be administered once per week, and even once every 2 weeks in maintenance patients [15,16].

Here, we report on an open-label, phase III study to assess the efficacy and safety of epoetin delta, administered subcutaneously once, twice or three times per week for the treatment of anaemia in haemodialysis, peritoneal dialysis and predialysis patients with CKD.

## Methods

### Patients

Patients with CKD (aged 18 years or older) and a medical history of anaemia (defined as haemoglobin < 11 g/dL) were eligible to enter the study. Predialysis patients had serum creatinine > 2 mg/dL (176.8 μmol L^-1^) or creatinine clearance rates of < 45 mL/min (by either 24-h urine collection or Cockcroft-Gault formula), equating to CKD stage 3 or 4. All patients had haemoglobin levels in the range of 9.6–12.4 g/dL for the 2 weeks before entering the study. In the 30 days before entering the study haemodialysis patients were receiving an epoetin (epoetin alfa or beta) subcutaneously, two or three times per week, while peritoneal dialysis patients and those not on dialysis were receiving an epoetin subcutaneously at least once per week. During this 30-day period the dose of the epoetin had not changed by > 50% (either increase or decrease).

Patients had to have serum ferritin ≥ 90 ng/mL and transferrin saturation ≥ 18% at the prestudy measurement. Sufficient iron to maintain serum ferritin ≥ 100 ng/mL and transferrin saturation ≥ 20% was specifically required during the study. If iron status measurements fell short of these criteria then patients received intravenous iron supplementation of at least 1000 mg, over a maximum of 10 weeks. For haemodialysis patients, iron was administered within the first 90 minutes of the dialysis session. At study centres in France oral iron supplementation was attempted before use of the intravenous route for predialysis and peritoneal dialysis patients, and for haemodialysis patients intravenous iron therapy was initiated within the first few minutes of the dialysis session.

Exclusion criteria included uncontrolled hypertension, concomitant illness that could reduce life expectancy to < 12 months, thrombocytopenia (platelet count < 75 000/mm^3^), impaired hepatic function, previous treatment with epoetin delta, and pregnancy or breastfeeding at the prestudy screening. Also excluded were women of child-bearing potential not using effective contraceptive methods.

The study was performed in accordance with the Declaration of Helsinki and was approved by local ethics committees at the individual study centres. All patients gave written informed consent before undergoing any study related procedures.

### Study Design

This was a multicentre, open-label, uncontrolled study to assess the safety and efficacy of subcutaneously administered epoetin delta over 1 year. The study was carried out at sites in the USA and Europe between 1998 and 2000.

Epoetin delta was administered subcutaneously once, twice or three-times per week, at the same frequency as patients received before entering the study. The initial dose of epoetin delta was identical to the last dose of epoetin the patient received before entering the study. This dose was maintained for 2 weeks with subsequent titration to maintain haemoglobin at 10–12 g/dL, in keeping with the European Best Practice Guidelines in place at the time of the study [17]. Titration was as follows: dose was increased by 50% if haemoglobin dropped below 10 g/dL or decreased by 25% if haemoglobin was above 12 g/dL. Dose was also altered if a patient's dry weight changed by more than 5 kg. Dose adjustment based on haemoglobin level was only performed once in every 4-week period.

### Endpoints and analysis

Haematological parameters were assessed weekly for the the first 12 weeks of the study and thereafter every 4 weeks. The primary efficacy endpoint was average haemoglobin over Weeks 12, 16, 20 and 24. Efficacy analyses were performed on data from a modified intent-to-treat (mITT) population consisting of all patients who had a valid haemoglobin measurement at baseline and at least one haemoglobin measurement during Weeks 12, 16, 20 or 24. An analysis of the primary endpoint was also carried out in the ITT population using last observation carried forward and first observation carried back techniques. In addition, an adjusted average haemoglobin was calculated with an ANCOVA model including parameters for dialysis type, pooled centre, and covariates of baseline haemoglobin and baseline epoetin dose.

Secondary analyses included: epoetin delta dose levels; descriptive statistics of haemoglobin and haematocrit levels; the proportion of patients' haemoglobin values over 10 g/dL and the proportion of patients' haematocrit values over 30% during Weeks 12–24; and haematological parameters and dosing over the 52 weeks of the study.

Safety assessments were conducted by recording and monitoring adverse events at each visit. Laboratory safety assessments were carried out every 4 weeks, and physical examinations and ECG at baseline, mid-study and at the end of the study (or early withdrawal). Anti-erythropoietin antibodies were screened for by validated ELISA (Quintiles Inc., Kansas City, MO) on samples collected at baseline and then every 4 weeks for the duration of the trial.

## Results

### Patients

**Figure 1:**
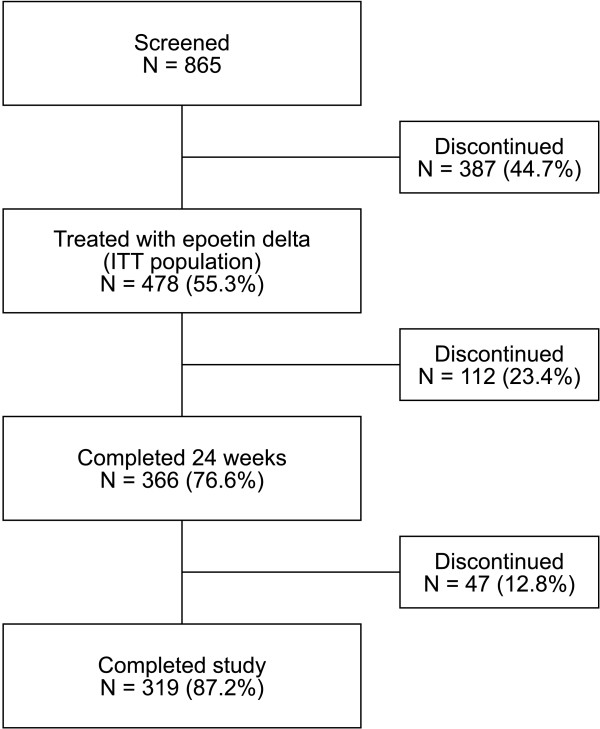
**Patient flow through the study**.

Of 865 patients screened, 478 met the inclusion criteria and entered the study (Figure 1), forming the ITT population. All patients in the ITT population received at least one dose of epoetin delta and were included in the safety-evaluable population. The mITT population consisted of 411 (86.0%) patients. This decrease from the ITT population is not surprising, given that criteria for inclusion in this group required completion of 12 weeks of treatment. The majority of haemodialysis patients received epoetin delta three-times or twice per week, while the majority of peritoneal dialysis and predialysis patients received epoetin delta once per week (Table 1). Baseline demographics for the mITT population are shown by both dialysis type and frequency of epoetin delta administration (Table 2).

**Table 1:** Frequency of administration by dialysis type and total patients (mITT population; N = 411)

	Number (%) of patients			
**Frequency of administration**	Predialysis (n = 32)	Haemodialysis (n = 288)	Peritoneal dialysis (n = 91)	Total (N = 411)

3× per week	0	152 (52.8)	6 (6.6)	158 (38.4)

2× per week	2 (6.3)	131 (45.5)	17 (18.7)	150 (36.5)

1× per week	30 (93.8)	5 (1.7)	68 (74.7)	103 (25.1)

**Table 2:** Baseline demographics by frequency of administration, dialysis type and total patients (mITT population; N = 411)

	Number (%) of patients (except as noted)						
	**By frequency of administration**			**By dialysis type**			

	3× weekly (n = 158)	2× weekly (n = 150)	1× weekly (n = 103)	Predialysis (n = 32)	Haemo-dialysis (n = 288)	Peritoneal dialysis (n = 91)	Total (N = 411)

**Sex**							

Male	101 (63.9)	82 (54.7)	50 (48.5)	13 (40.6)	175 (60.8)	45 (49.5)	233 (56.7)

Female	57 (36.1)	68 (45.3)	53 (51.5)	19 (59.4)	113 (39.2)	46 (50.5)	178 (43.3)

**Age (Years)**							

Mean (± SD)	59.4 (15.9)	60.5 (15.5)	56.7 (14.4)	63.9 (12.4)	60.7 (15.6)	52.4 (14.0)	59.2 (15.42)

Median (range)	61 (19–87)	64 (19–85)	58 (26–86)	66 (29–86)	64 (19–87)	53 (24–86)	61 (19–87)

**Race**							

Caucasian	110 (69.6)	111 (74.0)	64 (62.1)	26 (81.3)	212 (73.6)	47 (51.6)	285 (69.3)

Black	41 (25.9)	34 (22.7)	32 (31.1)	6 (18.8)	67 (23.3)	34 (37.4)	107 (26.0)

Asian or Oriental	2 (1.3)	0 (0.0)	3 (2.9)	0 (0.0)	1 (0.3)	4 (4.4)	5 (1.2)

Multiracial	5 (3.2)	5 (3.3)	4 (3.9)	0 (0.0)	8 (2.8)	6 (6.6)	14 (3.4)

The most common primary diagnoses for the causes of CKD were hypertensive nephrosclerosis (118/411; 28.7%) and diabetic nephropathy (97/411; 23.6%). Overall 34.5% (142/411) of patients had a medical history of either Type 1 or Type 2 diabetes.

Overall mean ± SD haemoglobin at baseline was 11.1 ± 0.9 g/dL (mean haematocrit of 35.0 ± 3.0%) and haemoglobin levels were similar across all dialysis types and administration frequencies (Table 3).

**Table 3:** Haemoglobin levels by frequency of administration, dialysis type and total patients (mITT population; N = 411)

	Average haemoglobin (g/dL) (mean ± SD)	
	
Population by:	Baseline	Weeks 12–24
**Frequency of administration**		

3× per week (n = 158)	11.0 ± 0.9	11.2 ± 1.1

2× per week (n = 150)	11.1 ± 0.8	11.3 ± 1.1

1× per week (n = 103)	11.1 ± 0.9	11.5 ± 1.2

**Dialysis type**		

Predialysis (n = 32)	10.9 ± 0.8	11.3 ± 1.2

Haemodialysis (n = 288)	11.1 ± 0.8	11.2 ± 1.1

Peritoneal dialysis (n = 91)	11.2 ± 0.9	11.6 ± 1.1

**Total (N = 411)**	11.1 ± 0.9	11.3 ± 1.1

Of those patients receiving study medication, 366/478 (76.6%) completed 24 weeks of treatment and 319/478 (66.7%) completed the study. The most common reasons for withdrawal in the first 24 weeks were: red blood cell transfusion (27/112; 24%); patient did not wish to continue (13/112; 11.6%) and death (13/112; 11.6%). In addition 19 patients in Europe were withdrawn because of a sterility test failure on a batch of study drug. Subsequent investigation indicated that no contamination of drug substance was present and there was a high probability that external contamination of the cap was responsible for the test failure. Other reasons for discontinuation included: missed more than six doses (n = 8); kidney transplant (n = 8); patient no longer met entry criteria (n = 7); adverse event (n = 6); patient transferred to another facility (n = 3); patient not previously prescribed an epoetin according to protocol for dialysis type (n = 3); miscellaneous (chemotherapy, surgery, investigator decision [n = 3]) and screening results not received in time (n = 2). The most common reasons for withdrawal during Weeks 24–52 were death (15/366; 4.1%), worsening adverse event (9/366; 2.5%), patient did not wish to continue (8/366; 2.2%), red blood cell transfusion (4/366; 1.1%) and kidney transplant (4/366; 1.1%).

### Efficacy assessments

**Figure 2:**
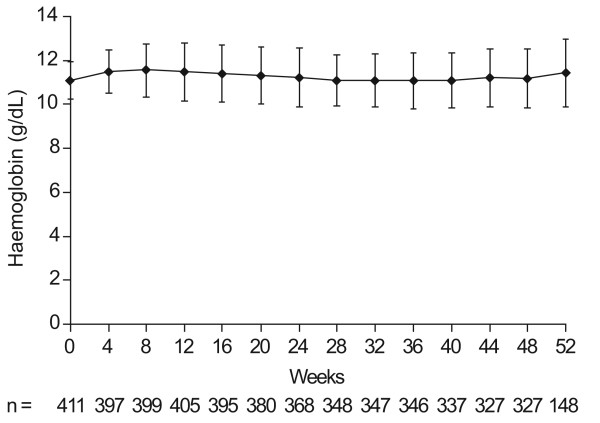
**Mean (± SD) haemoglobin during the 52 weeks of study**.

Overall mean ± SD haemoglobin level for Weeks 12–24 was 11.3 ± 1.1 g/dL, with mean haemoglobin levels maintained at 10–12 g/dL (within the target range) irrespective of dialysis type or administration frequency in the mITT population (Table 3). A similar overall value was obtained in the ITT population (11.3 ± 1.1 g/dL). The adjusted average level was 11.1 ± 0.1 g/dL, indicating that dialysis type, baseline haemoglobin level and baseline dose did not greatly affect the results. Mean ± SD haematocrit was 36.4 ± 3.7% over Weeks 12–24, and mean haematocrit during this period was > 36% for all dialysis subgroups and administration frequencies (data not shown). Over Weeks 12–24, 83.9% of haemoglobin measurements and 92.3% of haematocrit measurements were above the predefined lower limits of 10 g/dL and 30% respectively. Analyses of haemoglobin for the 52-week duration of the study, showed levels were maintained at 10–12 g/dL (Figure 2).

The average weekly doses over Weeks 12–24 and Weeks 37–52 are shown in Table 4, both by dialysis type and frequency of administration. Median dose was lowest for predialysis patients and highest for haemodialysis patients over Weeks 12–24. There were no marked differences in dose between Weeks 12–24 and Weeks 37–52. Mean and median weekly dose generally decreased with decreasing administration frequency, though the case mix in the three groups was different.

**Table 4:** Average weekly doses by frequency of administration, dialysis type and total patients (mITT population; N = 411)

	Average weekly doses (IU/kg)					
**Population by**	**Weeks 12–24**			**Weeks 37–52**		

**Frequency of administration**	**n (%)**	**Mean ± SD**	**Median (Range)**	**n (%)**	**Mean ± SD**	**Median (Range)**

3× per week	158 (100.0)	103.5 ± 88.0	77.9 (13.3–804.0)	126 (79.8)	114.4 ± 130.7	90.5 (0.0–1293.6)

2× per week	150 (100.0)	76.9 ± 58.5	62.2 (11.5–417.5)	131 (87.3)	79.2 ± 68.6	62.01 (2.5–436.5)

1× per week	103 (100.0)	65.9 ± 57.8	48.8 (2.3–335.0)	88 (85.4)	80.9 ± 96.8	47.5 (0.0–622.6)

**Dialysis type**						

Predialysis	32 (100.0)	87.7 ± 73.5	56.0 (19.1–335.0)	26 (81.2)	122.6 ± 133.3	73.2 (14.9–622.6)

Haemodialysis	288 (100.0)	82.3 ± 57.9	66.5 (2.3–396.0)	242 (84.0)	90.5 ± 102.6	69.0 (0.0–1293.6)

Peritoneal dialysis	91 (100.0)	89.8 ± 107.3	60.4 (12.2–804.0)	77 (84.6)	88.8 ± 92.4	61.9 (4.4–436.5)

**Total**	411 (100.0)	84.4 ± 72.7	64.8 (2.3–804.0)	345 (83.9)	92.5 ± 103.1	66.0 (0.0–1293.6)

### Safety and tolerability

Overall 86.8% (415/478) of patients experienced an adverse event during treatment, in line with the levels expected considering the baseline disease characteristics of this population. The most commonly reported adverse events were upper respiratory tract infection (19.5%), infection (18.2%), hypotension (16.7%), headache (14.4%) and muscle cramps (13.6%). Overall, adverse events considered possibly related to treatment occurred in 12.3% (59/478) of patients (Table 5). The most commonly reported were hypertension and thrombosis. Serious adverse events (SAEs) were reported by 236/478 (49.4%) of patients, with rates similar across all dialysis types. SAEs considered possibly related to study medication occurred in 4.6% (22/478) of patients, and the majority of these were reported for patients undergoing haemodialysis (17/22; 77.3%). Adverse events leading to withdrawal from the study occurred in 3.1% (15/478) of patients, with six events considered possibly related to study medication (angio-oedema, hypertensive encephalopathy, hypertension, nausea, skin ulcers, pruritus).

**Table 5:** Adverse events (AE) considered possibly related to treatment and reported in ≥ 0.5% of patients (safety-evaluable population; N = 478)

AE	Number (%) of patients
Total	59 (12.3)

Hypertension	14 (2.9)

Thrombosis	9 (1.9)

Anaemia	5 (1.0)

Injection site pain	5 (1.0)

Laboratory test abnormal*	4 (0.8)

Erythrocyte abnormal	3 (0.6)

Headache	3 (0.6)

*Laboratory test abnormal was the coded term for haemoglobin elevation (n = 2) and iron deficiency (n = 2)

Mean changes in laboratory analytes, vital signs and body weight from baseline to endpoint were minimal and there was no tendency for epoetin delta to induce ECG changes. During the 52 weeks of this trial no patient developed anti-erythropoietin antibodies or pure red cell aplasia.

## Discussion

Results from this open-label study show that subcutaneously administered epoetin delta, the only human-cell-derived epoetin, was effective for the control of anaemia in this group of CKD patients (predialysis, peritoneal dialysis and haemodialysis) over 52 weeks. During the primary period of assessment (Weeks 12–24) haemoglobin levels were maintained within the target range of 10–12 g/dL, in line with current recommendations [6]. Over 80% of haemoglobin and 90% of haematocrit measurements were above the predefined lower limits of 10 g/dL and 30% respectively. A switch from prestudy therapy with subcutaneous epoetin alfa to subcutaneous epoetin delta was associated with continued control of haemoglobin levels for up to 1 year, across all dialysis types and administration frequencies of once, twice and three times per week. Epoetin delta was well tolerated for the duration of the study and no patient developed anti-erythropoietin antibodies or pure red cell aplasia.

The data demonstrate that epoetin delta is an acceptable alternative to other epoetins. It would appear that patients can be transitioned from other epoetins to epoetin delta at the same dose with no loss of control of anaemia. As noted previously, epoetin delta differs from other ESAs in that it is produced in a human cell line. It is not known whether this leads to any clinical advantages, but in theory it is possible that a different glycosylation pattern may have an impact on the pleiotropic effects of the agent. This requires further investigation.

Our study has some limitations. The study was not blinded and did not include a control group and therefore results could possibly be due to factors other than treatment with epoetin delta. As entry criteria resulted in a study population that was previously responsive to epoetin treatment, our data provide no information on whether some patients may respond better to epoetin delta. It should also be noted that this study was completed before recent evidence regarding the potential harmful effects of fully correcting haemoglobin levels. From our data, we can not draw any conclusion as to whether epoetin delta was associated with any risk or benefit when associated with relatively high haemoglobin levels.

Further studies are underway to investigate potential benefits arising from the human-cell production of epoetin delta.

## Conclusion

In this 52-week trial subcutaneously administered epoetin delta was shown to be an effective agent for the management of anaemia in patients with CKD. Predialysis patients and patients requiring peritoneal dialysis or haemodialysis were all able to maintain haemoglobin levels within the specified range, without marked changes in dosing regimen. Epoetin delta was well tolerated for treatment of up to 1 year and there was no evidence that it was capable of eliciting an immune response.

## Competing interests

This study was sponsored by Hoechst Marion Roussel. The article processing charge will be financed by Shire plc. U Frei has received honoraria from Shire for consultancy and presentations. JTC Kwan and BS Spinowitz declare that they have no competing interests.

## Authors' contributions

All authors enrolled patients in the study, contributed substantially to the development of the manuscript, and have read and approved the final paper.

## Pre-publication history

The pre-publication history for this paper can be accessed here:

http://www.biomedcentral.com/1471-2369/10/5/prepub
